# Efficacy of antihyperglycemic therapies on cardiovascular and heart failure outcomes: an updated meta-analysis and meta-regression analysis of 35 randomized cardiovascular outcome trials

**DOI:** 10.1186/s12933-023-01773-z

**Published:** 2023-03-19

**Authors:** Masashi Hasebe, Satoshi Yoshiji, Yamato Keidai, Hiroto Minamino, Takaaki Murakami, Daisuke Tanaka, Yoshihito Fujita, Norio Harada, Akihiro Hamasaki, Nobuya Inagaki

**Affiliations:** 1 Department of Diabetes and Endocrinology, Medical Research Institute KITANO HOSPITAL, PIIF Tazuke-Kofukai, Osaka, Japan; 2 Department of Diabetes, Endocrinology and Nutrition, Kyoto University Graduate School of Medicine, 54 Kawahara-cho, Shogoin, Sakyo-ku, Kyoto, 606-8507 Japan; 3 Department of Human Genetics, McGill University, Montréal, QC Canada; 4 Kyoto-McGill International Collaborative Program in Genomic Medicine, Graduate School of Medicine, Kyoto University, Kyoto, Japan

**Keywords:** Type 2 diabetes, Cardiovascular outcome trials, Cardiovascular events, Heart failure, Glycemic control, Bodyweight control, Meta-analysis

## Abstract

**Background:**

Effects of antihyperglycemic therapies on cardiovascular and heart failure (HF) risks have varied widely across cardiovascular outcome trials (CVOTs), and underlying factors remain incompletely understood. We aimed to determine the relationships of glycated hemoglobin (HbA1c) or bodyweight changes with these outcomes in all CVOTs of antihyperglycemic therapies.

**Methods:**

We searched PubMed and EMBASE up to 25 January 2023 for all randomized controlled CVOTs of antihyperglycemic therapies reporting both major adverse cardiovascular events (MACE) and HF outcomes in patients with type 2 diabetes or prediabetes. We performed meta-regression analyses following random-effects meta-analyses to evaluate the effects of HbA1c or bodyweight reductions on each outcome.

**Results:**

Thirty-five trials comprising 256,524 patients were included. Overall, antihyperglycemic therapies reduced MACE by 9% [risk ratio (RR): 0.91; 95% confidence interval (CI) 0.88–0.94; *P* < 0.001; *I*^*2*^ = 36.5%]. In meta-regression, every 1% greater reduction in HbA1c was associated with a 14% reduction in the RR of MACE (95% CI 4–24; *P* = 0.010), whereas bodyweight change was not associated with the RR of MACE. The magnitude of the reduction in MACE risk associated with HbA1c reduction was greater in trials with a higher baseline prevalence of atherosclerotic cardiovascular disease. On the other hand, antihyperglycemic therapies showed no overall significant effect on HF (RR: 0.95; 95% CI 0.87–1.04; *P* = 0.28; *I*^*2*^ = 75.9%). In a subgroup analysis based on intervention type, sodium-glucose cotransporter-2 inhibitors (SGLT2i) conferred the greatest HF risk reduction (RR: 0.68; 95% CI 0.62–0.75; *P* < 0.001; *I*^*2*^ = 0.0%). In meta-regression, every 1 kg bodyweight reduction, but not HbA1c reduction, was found to reduce the RR of HF by 7% (95% CI 4–10; *P* < 0.001); however, significant residual heterogeneity (*P* < 0.001) was observed, and SGLT2i reduced HF more than could be explained by HbA1c or bodyweight reductions.

**Conclusions:**

Antihyperglycemic therapies reduce MACE in an HbA1c-dependent manner. These findings indicate that HbA1c can be a useful marker of MACE risk reduction across a wide range of antihyperglycemic therapies, including drugs with pleiotropic effects. In contrast, HF is reduced not in an HbA1c-dependent but in a bodyweight-dependent manner. Notably, SGLT2i have shown class-specific benefits for HF beyond HbA1c or bodyweight reductions.

**Supplementary Information:**

The online version contains supplementary material available at 10.1186/s12933-023-01773-z.

## Introduction

People with type 2 diabetes are at high risk of developing cardiovascular events, including cardiovascular death, coronary heart disease, stroke, and heart failure (HF) [1]. To date, various clinical trials have investigated the efficacy of antihyperglycemic therapies on cardiovascular outcomes, and some have provided evidence of a significant reduction in the risk of major adverse cardiovascular events (MACE) and/or hospitalization for HF in patients with type 2 diabetes or prediabetes [2]. Although it is presumed that the cardiovascular protection conferred by antihyperglycemic therapies is attributable to various variables, it is unclear which variables affect the development of cardiovascular diseases in chronic glycemia management.

Antihyperglycemic therapies lower blood glucose levels through various mechanisms to mitigate hyperglycemia symptoms and diabetes-related complication risk. Many cardiovascular outcome trials have shown a significant difference in glycemic control between the intervention (medication or intensive care) and control (placebo or standard care) groups during the observation period, even when they were designed to achieve “glycemic equipoise” between trial arms. Moreover, the effects of antihyperglycemic therapies on total bodyweight have varied substantially across previous clinical trials.

Thus far, various pooled analyses of cardiovascular outcome trials have been reported that have examined the risk modulation of cardiovascular and HF outcomes conferred by alterations in blood glucose levels and bodyweight. For instance, in a previous meta-analysis of 30 large-scale cardiovascular outcome trials that was published in 2020, various glucose-lowering drugs or strategies that significantly reduced the glycated hemoglobin (HbA1c) level (> 0.01%) also reduced MACE risk, but they did not ameliorate HF risk compared with standard care or placebo [3]. In that study, the meta-regression analysis showed that bodyweight reduction was associated with HF risk reduction. In other meta-analyses of cardiovascular outcome trials involving newer antihyperglycemic medications (such as dipeptidyl-peptidase-4 inhibitors [DPP-4i], glucagon-like peptide-1 receptor agonists [GLP-1RA], and sodium-glucose cotransporter-2 inhibitors [SGLT2i]), significant associations were observed between improvements in glycemic control and the reduction of MACE risk [4–7], whereas no association was identified between glycemic improvement and the risk of HF [5–7]. Additionally, despite the acknowledged association between obesity and an increased risk of atherosclerotic cardiovascular disease (ASCVD) [8], the meta-analysis of cardiovascular outcome trials involving GLP-1RA revealed no correlation between bodyweight reduction and the risk of MACE [4]. However, whether and to what extent blood glucose lowering and bodyweight reduction are associated with cardiovascular and HF benefits has not been comprehensively studied and updated to include all trials of antihyperglycemic therapies completed before and after the establishment of the Food and Drug Administration (FDA) guidelines in 2008 [9]. The trials completed before the establishment of the FDA guidelines include the United Kingdom Prospective Diabetes Study (UKPDS), and those completed after the establishment of the FDA guidelines include newer studies of DPP-4i, GLP-1RA, and SGLT2i.

Recently, the results of more cardiovascular outcome trials with newer antihyperglycemic medications have become available. Therefore, we performed a comprehensive, updated meta-analysis and meta-regression analysis of 35 large-scale cardiovascular outcome trials of antihyperglycemic drugs reporting MACE and HF outcomes published both before and after the establishment of the FDA guidelines in 2008. We aimed to explore the relationships between blood glucose lowering or bodyweight reduction and MACE and HF risks, thereby delineating the potential contribution of blood glucose lowering or bodyweight reduction per se to cardiovascular and HF benefits.

## Method

### Search strategies and selection criteria

The protocol of this study has been registered at the International Prospective Register of Systematic Reviews (PROSPERO) (registration number: CRD42022299075). This study was performed in accordance with the Preferred Reporting Items for Systematic Reviews and Meta-analysis (PRISMA) guidelines [10]. We followed the eligibility criteria previously established [3]: (i) randomized controlled trials with an enrollment of a minimum of 1000 adults with type 2 diabetes or prediabetes, (ii) compared an antihyperglycemic drug, an intensive glycemic control strategy, or a lifestyle intervention strategy with controls (placebo, standard care, or an active control agent), (iii) reported MACE and HF as outcomes of interest, (iv) a follow-up period of at least one year, and (v) achieved an HbA1c difference greater than 0.01% between trial arms. Trials were excluded if they reported an achieved HbA1c difference of ≤ 0.01%, or if they did not report the difference in achieved HbA1c between the trial arms. We also excluded trials if a multifactorial intervention or non-glycemic medication were examined.

**Fig. 1:**
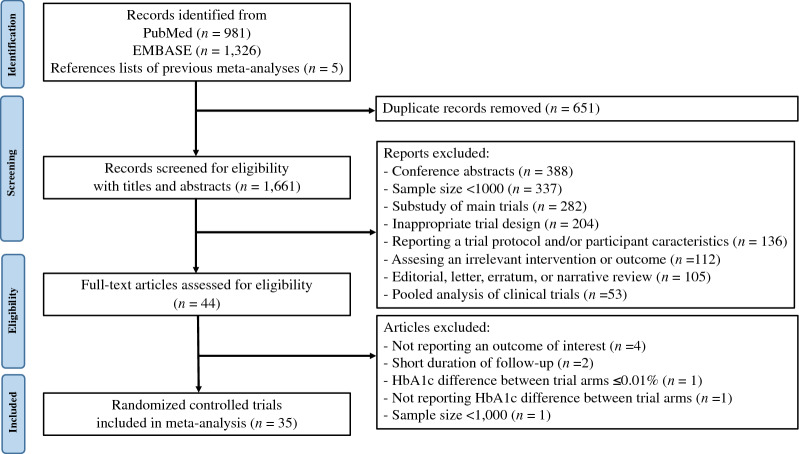
Preferred Reporting Items for Systematic Reviews and Meta-Analysis (PRISMA) flow diagram for study selection

We conducted a literature search on PubMed and EMBASE databases from their inception until 25 January 2023 without language restriction to find relevant studies using the search strategies as follows: (type 2 diabetes OR prediabetes) AND (randomized OR randomly) AND (cardiovascular OR macrovascular OR MACE OR heart failure) AND (antihyperglycemic OR antidiabetic OR intensive glucose control OR intensive blood glucose control OR intensive blood-glucose control OR intensive glucose lowering OR lifestyle intervention OR biguanide* OR sulfonylurea* OR glinide* OR meglitinide* OR peroxisome proliferator-activated receptor* OR thiazolidinedione OR α-glucosidase inhibitor* OR dipeptidyl peptidase 4 OR glucagon-like peptide 1 OR glucagon-like peptide-1 OR sodium-glucose cotransporter 2). We also manually searched the reference lists of previous meta-analyses of cardiovascular outcome trials to identify potentially relevant studies. The PRISMA flow diagram is shown in Fig. 1.

### Data extraction and quality assessment

From the eligible trials, we extracted data on study characteristics, baseline characteristics of participants, antihyperglycemic regimens used, control regimens used, mean differences in the achieved HbA1c and bodyweight levels between trial arms, and outcomes of interest that included risk ratios (RRs) with 95% confidence intervals (CIs). We extracted data from primary trial results and their accompanying supplementary materials as the primary data source. To determine the achieved differences in HbA1c or bodyweight levels between the trial arms, we used the time-weighted least squares mean difference over the course of follow-up, at the end of follow-up, or at one year of follow-up. We used the first available follow-up data if none of these values were reported. We used version 2 of the Cochrane Risk of Bias Tool to evaluate the risk of bias in the eligible studies [11]. MH and YK independently conducted the literature search and data extraction. The results were compared, and any discrepancies were resolved by consensus or with input from a third independent reviewer (SY).

### Data analysis

The primary outcome was the efficacy of antihyperglycemic therapies on HF and MACE risks (defined as a composite of cardiovascular death, non-fatal myocardial infarction [MI], or non-fatal stroke). We determined the relationships between HbA1c reduction or bodyweight change and HF or MACE risk using meta-regression. If the trials did not report MACE according to our aforementioned definition, we used one of the following alternative definitions: death from cardiovascular or undetermined causes, non-fatal MI, or non-fatal stroke; cardiovascular death, non-fatal MI, or non-fatal ischemic stroke; all-cause death, non-fatal MI, or non-fatal stroke; cardiovascular death, non-fatal MI, non-fatal stroke, or other atherothrombotic events; all-cause death, non-fatal MI, non-fatal stroke, or other atherothrombotic events; fatal and non-fatal MI or stroke; or fatal and non-fatal MI. Detailed definitions of HF and MACE outcomes in each trial are displayed in Additional file 1: Table S1.

For the meta-analysis, pooled RRs with 95% CIs for MACE and HF outcomes were calculated using a random-effects model with the inverse variance method [12]. The between-trial variance was estimated using the DerSimonian–Laird estimator [13]. Heterogeneity among trials was evaluated using Cochran’s Q test and Higgins’s *I*^*2*^ statistics [14]. Thresholds defining the magnitude of heterogeneity based on the *I*^*2*^ index were low (≤ 25%), moderate (26–50%), and high (> 50%) [15]. Subgroup random-effects meta-analysis was performed based on a type of intervention (an intensive glycemic control strategy or each drug class). Additionally, we evaluated publication bias by funnel plots and Egger’s test [16].

For the meta-regression analysis with a mixed-effects model, we analyzed the association between the differences in the achieved HbA1c or bodyweight difference and the corresponding estimated log RR [Ln(RR)] of MACE and HF outcomes. To obtain the relative RR reduction of each outcome for every 1% HbA1c reduction or every 1 kg bodyweight reduction, we used the following formula with the regression coefficient (slope) in the meta-regression (Additional file 1: Fig. S1):

$${\text{Relative RR reduction }}\left( {\text{\% }} \right){\text{ for every 1\% HbA1c reduction or 1 kg bodyweight reduction}}\,{ = }\,(1 \, {-}e^{{{\text{slope}}}} ) \times 100$$

We used the restricted maximum likelihood as an estimator [17]. If a significant association was identified in the primary meta-regression analyses, we conducted trial-level subgroup meta-regression, stratified by (1) type of intervention (an intensive glycemic control strategy or each drug class) and (2) baseline prevalence of ASCVD (≥ 70% vs. < 70%). Trials without reported baseline proportions of patients with ASCVD were excluded from the latter subgroup analysis. Sensitivity meta-regression was performed to adjust for trial-level mean age, proportion of female participants, and person-years (calculated as the number of participants multiplied by the median follow-up duration in years, divided by 1000) to assess the consistency of the significant association found in the primary analysis.

Results with two-sided *P*-values less than 0.05 were considered significant for the pooled RR meta-analysis and meta-regression analysis. *P*-values less than 0.05 in Cochran’s Q test were also considered significant. All analyses were performed using R statistical software (version 4.1.2; the R Foundation for Statistical Computing, Vienna, Austria) with the ‘meta’ (version 4.18.2) and ‘metafor’ (version 3.02) packages.

## Results

### Search results and baseline study characteristics

The initial search identified 1661 trials after removing duplicates. Screening for eligibility using titles and abstracts yielded 44 trials for detailed assessment, of which nine trials did not meet the inclusion criteria and were excluded (Fig. 1, Additional file 1: Table S2). Trials excluded through the full-text assessment included the CAROLINA trial, which compared linagliptin with glimepiride and showed no significant difference in both the achieved HbA1c and the MACE/ HF outcomes [18]. Therefore, 35 trials comprising 256,524 patients were included in the meta-analysis [19–53]. Among the 35 included trials, four assessed an intensive glycemic control strategy, one assessed intensive lifestyle intervention focusing on weight loss, one assessed insulin glargine (long-acting insulin analog), one assessed acarbose (α-glucosidase inhibitor), eight assessed peroxisome proliferation-activated receptor (PPAR) agonists, five assessed DPP-4i, nine assessed GLP-1RA, and six assessed SGLT2i. The main characteristics of the included trials are shown in Table 1. The mean follow-up duration was 1.3–10.0 years, and the average age of the patients was 53.3–69.0 years. A total of 164,276 of 248,306 (66.2%) assessable patients had established atherosclerotic cardiovascular disease, and 30,708 of 229,343 (13.4%) assessable patients had a history of HF at baseline. Although the trials designed as open-label had a high risk of bias in the domain of deviations from intended interventions because of the inability to blind the intervention, the evaluation of eligible trials showed no obvious risk of bias in most other domains. The risk of bias of each eligible trial is summarized in Additional file 1: Table S3. A visual inspection of funnel plots and the results of Egger’s test for outcomes of interest both indicated no evidence of publication bias (*P* Egger’s test = 0.42 and 0.79 for MACE and HF outcome, respectively); however, the ADOPT and DREAM trials, both of which investigated the efficacy of rosiglitazone, were observed as outliers in the funnel plot for MACE (Additional file 1: Fig. S2) [21, 22]. DREAM was also identified as an outlier in the funnel plot for HF (Additional file 1: Fig. S2) [22].

**Table 1:** Key characteristics of the included trials

Trial	Year	Participants, *n*	Intervention	Control	Median follow-up, years	Mean age (SD), years	Mean diabetes duration, years	Female, *n*	Mean BMI (SD), kg/m^2^	Baseline CVD, *n*	Baseline HF, *n*	Mean baseline HbA1c (SD), %	Mean HbA1c reduction, %	Mean bodyweight change, kg
UKPDS 33	1998	3867	IGC with sulfonylurea or insulin (target fasting blood glucose < 6 mmol/L)	Standard care (diet; target fasting blood glucose < 15 mmol/L)	10.0	53.3 (8.6)	0.0^a^	1508 (39%)	27.5 (5.2)	NR	NR	7.1 (1.5)	0.90	+ 3.10
PROactive	2005	5238	PPAR agonists (pioglitazone 15 to 45 mg/day)	Placebo	2.9	61.7 (7.7)	8.0^a^	1775 (34%)	30.8 (0.5)	5238 (100%)	NR	8.0 (NR)	0.50	+ 4.00
ADOPT	2006	4351	PPAR agonists (rosiglitazone 4 to 8 mg/day)	Metformin (500 to 2000 mg) or glyburide (2.5 to 15 mg)	4.0	56.9 (10.1)	1.5	1840 (42%)	32.2 (6.4)	NR	0 (0%)	7.4 (1.0)	0.13 (vs. metformin); 0.42 (vs. glyburide)	+ 6.90 (vs. metformin); + 2.50 (vs. glyburide)
DREAM	2006	5269	PPAR agonists (rosiglitazone 4 to 8 mg/day)	Placebo	3.0	54.7 (10.9)	0.0	3120 (59%)	30.9 (5.6)	0 (0%)	0 (0%)	NR	0.50	+ 2.20
ACCORD	2008	10,251	IGC (target HbA1c < 6.0%)	Standard care (target HbA1c 7.0–7.9%)	10.0	62.2 (6.8)	10.0	3952 (39%)	32.2 (5.5)	3608 (35%)	497 (5%)	8.3 (1.1)	1.10	+ 3.10
ADVANCE	2008	11,140	IGC with gliclazide and other drugs as required (target HbA1c < 6.0%)	Standard care (target HbA1c 7.0–7.9%)	5.0	66.0 (6.0)	7.9	4735 (43%)	28.0 (5.0)	3590 (32%)	NR	7.5 (1.6)	0.67	+ 0.70
BARI 2D	2009	2368	Insulin-sensitization therapy with oral treatment	Insulin-provision therapy	5.3	62.4 (8.9)	10.4	701 (30%)	31.7 (5.4)	2368 (100%)	156 (6.6%)	7.7 (1.6)	0.50	− 1.80
RECORD	2009	4447	PPAR agonists (rosiglitazone 4 to 8 mg/day)	Metformin (at a maximum dose of 2550 mg) and sulfonylurea (glibenclamide at a maximun dose of 15 mg or equivalent for different preparations)	5.5	58.4 (8.3)	7.1	2154 (48%)	31.5 (4.8)	772 (17.4%)	21 (0.5%)	7.9 (0.7)	0.27	+ 4.70
VADT	2009	1791	IGC (treatment absolute difference in HbA1c ≤ 1.5%)	Standard care	5.6	60.4 (9.0)	11.5	52 (3%)	31.2 (3.5)	723 (40%)	NR	9.4 (2.0)	1.50	+ 4.05
ORIGIN	2012	12,537	Insulin glargine (target fasting blood glucose < 5.3 mmol/L)	Standard care	6.2	63.5 (7.9)	5.4	4386 (35%)	29.9 (5.3)	7378 (59%)	NR	6.4 (NR)	0.30	+ 2.10
EXAMINE	2013	5380	DPP-4i (alogliptin 25 mg/day)	Placebo	1.5	61.0 (10.0)	7.2^a^	1729 (32%)	28.3 (NR)	5380 (100%)	1501 (28%)	8.0 (1.1)	0.36	+ 0.06
Look AHEAD	2013	5145	Intensive lifestyle intervention	Standard care	9.6	58.8 (6.9)	5.0^a^	3063 (60%)	35.9 (5.9)	714 (14%)	NR	7.3 (1.2)	0.22	− 4.00
SAVOR-TIMI 53	2013	16,492	DPP-4i (saxagliptin 5 mg/day)	Placebo	2.1	65.1 (8.5)	10.3^a^	5455 (33%)	31.2 (5.6)	12,959 (79%)	2105 (13%)	8.0 (1.4)	0.20	− 0.10
AleCardio	2014	7226	PPAR agonists (aleglitazar 150 μg/day)	Placebo	2.0	61.0 (10.0)	8.6	1966 (27%)	28.7 (NR)	7226 (100%)	759 (11%)	7.8 (1.7)	0.60	+ 3.70
ELIXA	2015	6068	GLP-1RA (lixisenatide 20 μg/day)	Placebo	2.1	59.9 (9.7)	9.2	1861 (31%)	30.1 (5.6)	6068 (100%)	1358 (22%)	7.7 (1.3)	0.27	− 0.70
EMPA-REG OUTCOME	2015	7020	SGLT2i (empagliflozin 10 or 25 mg/day)	Placebo	3.1	63.1 (8.6)	57% > 10 years^b^	2004 (24%)	30.6 (5.3)	7020 (100%)	706 (10%)	8.1 (0.8)	0.57	− 2.00
TECOS	2015	14,671	DPP-4i (sitagliptin 100 mg/day)	Placebo	3.0	65.5 (8.0)	11.6	4297 (29%)	30.2 (5.6)	10,863 (74%)	2643 (18%)	7.2 (0.5)	0.29	− 0.05
IRIS	2016	3876	PPAR agonists (pioglitazone 30 mg/day)	Placebo	4.8	63.5 (10.6)	0.0	1338 (35%)	29.9 (10.5)	3876 (100%)	0 (0%)	5.8 (0.4)	0.06	+ 0.00
LEADER	2016	9340	GLP-1RA (liraglutide 1.8 mg/day)	Placebo	3.8	64.2 (7.2)	12.8	3337 (36%)	32.5 (6.3)	7598 (81%)	1667 (18%)	8.7 (1.6)	0.40	− 2.30
SUSTAIN-6	2016	3297	GLP-1RA (semaglutide 0.5 or 1.0 mg/week)	Placebo	2.1	64.6 (7.4)	13.9	1295 (39%)	32.8 (6.2)	2735 (83%)	777 (24%)	8.7 (1.5)	0.85	− 3.61
OMNEON	2017	4202	DPP-4i (omaligliptin 25 mg/week)	Placebo	1.8	63.6 (8.5)	12.1	1254 (30%)	31.3 (5.5)	4202 (100%)	641 (15%)	8.0 (0.9)	0.30	− 0.08
CANVAS Program	2017	10,142	SGLT2i (canagliflozin 100 or 300 mg/day)	Placebo	2.4	63.3 (8.3)	13.5	3633 (36%)	32.0 (5.9)	6656 (66%)	1461 (14%)	8.2 (0.9)	0.58	− 1.60
EXSCEL	2017	14,752	GLP-1RA (exenatide 2 mg/week)	Placebo	3.2	61.9 (9.4)	13.1	5603 (38%)	32.7 (6.4)	10,782 (73%)	2389 (16%)	8.1 (1.0)	0.53	− 1.27
ACE	2017	6522	α-GI (acarbose 50 mg three times/day)	Placebo	5.0	64.3 (8.1)	0.0	1762 (27%)	25.4 (3.1)	6522 (100%)	69 (1%)	5.9 (0.7)	0.07	− 0.64
TOSCA.IT	2017	3028	PPAR agonists (pioglitazone 15 to 45 mg/day)	Sulfonylurea (glibenclamide 5–15 mg or gliclazide 30–120 mg or glimepiride 2–6 mg)	4.8	62.3 (6.5)	8.5	1254 (41%)	30.3 (4.5)	335 (11%)	0 (0%)	7.7 (0.5)	0.24	+ 3.10
HARMONY Outcomes	2018	9463	GLP-1RA (albiglutide 30 or 50 mg/wek)	Placebo	1.5	64.1 (8.7)	14.1	2894 (31%)	32.3 (5.9)	9463 (100%)	1922 (20%)	8.7 (1.5)	0.52	− 0.83
DECLARE-TIMI 58	2019	17,160	SGLT2i (dapaglifrozin 10 mg/day)	Placebo	4.2	63.9 (6.8)	11.8	6422 (37%)	32.1 (6.0)	6974 (41%)	1724 (10%)	8.3 (1.2)	0.42	− 1.80
CARMELINA	2019	6979	DPP-4i (linagliptin 5 mg/day)	Placebo	2.2	65.9 (9.1)	14.7	2589 (37%)	31.4 (5.4)	4081 (58%)	1873 (27%)	7.9 (1.0)	0.36	− 0.15
CREDENCE	2019	4401	SGLT2i (canagliflozin 100 mg/day)	Placebo	2.6	63.0 (9.2)	15.8	1494 (34%)	31.3 (6.2)	2220 (50%)	652 (15%)	8.3 (1.3)	0.25	− 0.80
REWIND	2019	9901	GLP-1RA (dulaglutide 1.5 mg/week)	Placebo	5.4	66.2 (6.5)	10.5	4589 (46%)	32.3 (5.7)	3114 (31%)	853 (9%)	7.3 (1.1)	0.61	− 1.46
PIONEER 6	2019	3138	GLP-1RA (oral semaglutide 14 mg/day)	Placebo	1.3	66.0 (7.0)	14.9	1007 (32%)	32.3 (6.5)	2695 (85%)	288 (12%)	8.2 (1.6)	0.70	− 3.40
VERTIS CV	2020	8246	SGLT2i (ertugliflozin 5 or 15 mg/day)	Placebo	3.0	64.4 (8.1)	13.0	2477 (30%)	31.9 (5.4)	8246 (100%)	1958 (24%)	8.2 (1.0)	0.50	− 2.40
SCORED	2021	10,584	SGLT2i (sotagliflozin 200 or 400 mg/day)	Placebo	1.3	69 (63–74), 69 (63–74)^c^	NR	4754 (45%)	31.9 (28.1–36.2), 31.7 (28.0–36.1)^c^	5144 (49%)	3283 (31%)	8.3 (7.6–9.3), 8.3 (7.6–9.4)^c^	0.42	− 1.16
AMPLITUDE-O	2021	4076	GLP-1RA (efpeglenatide 4 or 6 mg/week)	Placebo	1.8	64.5 (8.2)	14.9	1344 (33%)	32.7 (6.2)	3650 (90%)	737 (18%)	8.9 (1.5)	1.24	− 2.60
FREEDOM-CVO	2022	4156	GLP-1RA (continuously infused exenatide 20 μg/day)	Placebo	1.3	63 (58–68), 63 (57–68)^c^	NR	1525 (37%)	32.4 (28.8–36.6), 31.9 (28.6–36.1)^c^	2036 (49%)^d^	668 (16%)	8.0 (7.2–9.3), 8.0 (7.2–9.2)^c^	0.84	− 4.24

*BMI* body mass index, *CVD* cardiovascular disease, *DPP-4i* dipeptidyl-peptidase-4 inhibitor, *GLP-1RA* glucagon-like peptide-1 receptor agonist, *HbA1c* glycated hemoglobin, *HF* heart failure, *IGC* intensive glycemic control, *NR* not reported, *PPAR* peroxisome proliferator-activated receptor, *SGLT2i* sodium-glucose co-transporter-2 inhibitor

^a^Median value

^b^Approximately 57% of the participants had more than 10 years of diabetes duration

^c^Continuous data (baseline age, BMI, and HbA1c) of SCORED and FREEDOM-CVO are separately presented as median (interquartile range) in intervention group and control group, respectively

^d^History of coronary artery disease

### Major adverse cardiovascular events

**Fig. 2:**
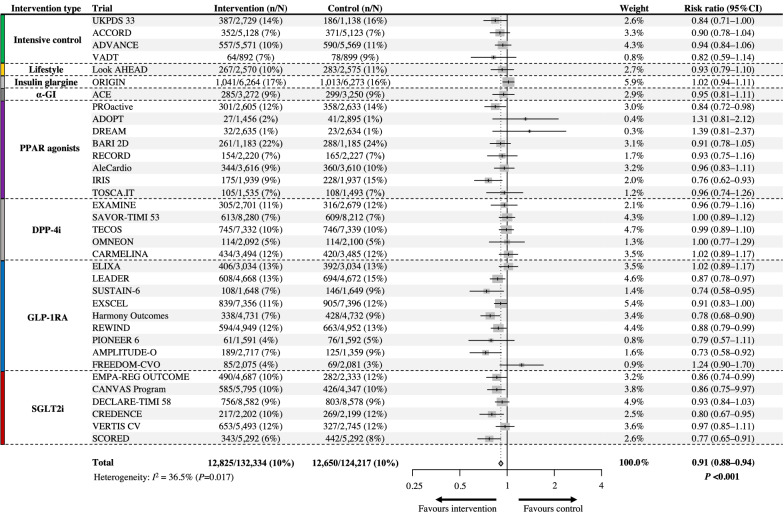
Efficacy of antihyperglycemic drugs on the risk of major adverse cardiovascular events (MACE). UKPDS 33, ACCORD, ADVANCE, VADT: trials comparing an intensive glycemic control strategy with standard care; Look AHEAD: a trial comparing intensive lifestyle intervention for weight loss with standard care; ORIGIN: a trial comparing insulin glargine with standard care; ACE: a trial comparing acarbose (α-glucosidase inhibitor [α-GI]) with placebo; PROactive, ADOPT, DREAM, BARI 2D, RECORD, AleCardio, IRIS, TOSCA.IT: trials comparing peroxisome proliferation-activated receptor (PPAR) agonists with placebo or active control drug; EXAMINE, SAVOR-TIMI 53, TECOS, OMNEON, CARMELINA: trials comparing dipeptidyl-peptidase-4 inhibitors (DPP-4i) with placebo; ELIXA, LEADER, SUSTAIN-6, EXSCEL, Harmony Outcomes, REWIND, PIONEER 6, AMPLITUDE-O, FREEDOM-CVO: trials comparing glucagon-like peptide-1 receptor agonists (GLP-1RA) with placebo; EMPAREG-OUTCOME, CANVAS-Program, DECLARE-TIMI 58, CREDENCE, VERTIS CV, SCORED: trials comparing sodium-glucose cotransporter-2 inhibitors (SGLT2i) with placebo

Overall, 25,475 patients (9.9%) experienced MACE outcomes during the follow-up period. In the pooled analysis of 35 trials, antihyperglycemic therapies decreased MACE risk by 9%, with moderate heterogeneity between trials (RR: 0.91; 95% CI 0.88–0.94; *P* < 0.001; *I*^*2*^ = 36.5%) (Fig. 2). In a subgroup random-effects meta-analysis based on the type of intervention, intensive glycemic control strategies (RR: 0.90; 95% CI 0.83–0.97; *P* = 0.008; *I*^*2*^ = 0.0%), PPAR agonists (RR: 0.91; 95% CI 0.84–0.97; *P* = 0.006; *I*^*2*^ = 25.2%), GLP-1RA (RR: 0.87; 95% CI 0.81–0.94; *P* = 0.001; *I*^*2*^ = 53.3%), and SGLT2i (RR: 0.88; 95% CI 0.82–0.94; *P* < 0.001; *I*^*2*^ = 28.1%) conferred a significantly lower risk of MACE to a similar extent, whereas the others showed null effects on MACE risk (Additional file 1: Fig. S3).

**Fig. 3:**
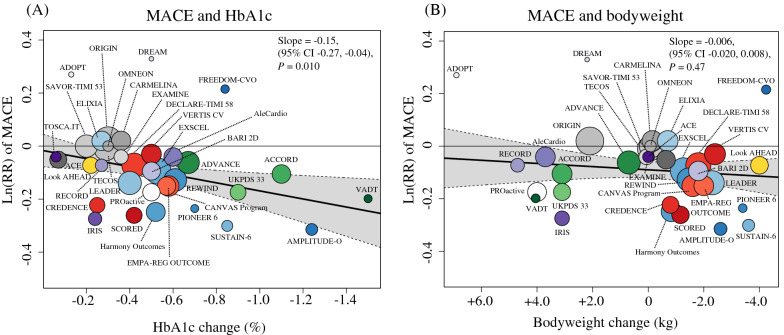
Association between the risk of major adverse cardiovascular events (MACE) and **A** HbA1c reduction or **B** bodyweight change. The thicker line shows meta-regression with 95% CI as shading. The circle size of each trial reflects the study weight. *HbA1c* glycated hemoglobin, *CI* confidence interval, *Ln(RR)* estimated log risk ratio

The univariate meta-regression analysis revealed a significant association between the HbA1c reduction from baseline and the Ln(RR) of MACE (slope: − 0.15; 95% CI − 0.27 to − 0.04; *P* = 0.010; variance explained: 52%) (Fig. 3A). Accordingly, every 1% greater reduction in HbA1c was associated with a 14% (95% CI 4–24) relative reduction in the RR of MACE. In contrast, bodyweight change from baseline was not significantly associated with the Ln(RR) of MACE (slope: − 0.006; 95% CI − 0.020 to 0.008; *P* = 0.41) (Fig. 3B).

We further evaluated the robustness of the relationship between HbA1c reduction and the risk of MACE through subgroup and sensitivity meta-regression analyses. In the stratified meta-regression by type of intervention, trials involving intensive glycemic control strategies, DPP-4i, and GLP-1RA showed a trend of reducing the Ln(RR) of MACE in association with a decrease in HbA1c; however, the relationship between HbA1c reduction and MACE risk was not statistically significant for all intervention types (Additional file 1: Table S4). In the stratified meta-regression based on the baseline prevalence of ASCVD (≥ 70% vs. < 70%), HbA1c reduction was associated with a decrease in the Ln(RR) of MACE for both subgroups. Notably, the decrease in MACE risk associated with HbA1c reduction was significant and greater in trials with ≥ 70% of patients with ASCVD at baseline (Additional file 1 Fig. S4). On the sensitivity meta-regression analysis with adjustment for multiple confounders (trial-level age, sex, and person-years), HbA1c reduction was significantly associated with the risk reduction of MACE, in agreement with the results of the main univariate analysis (slope: − 0.15; 95% CI − 0.26 to − 0.04; *P* = 0.008; variance explained: 89%).

### Heart failure

**Fig. 4:**
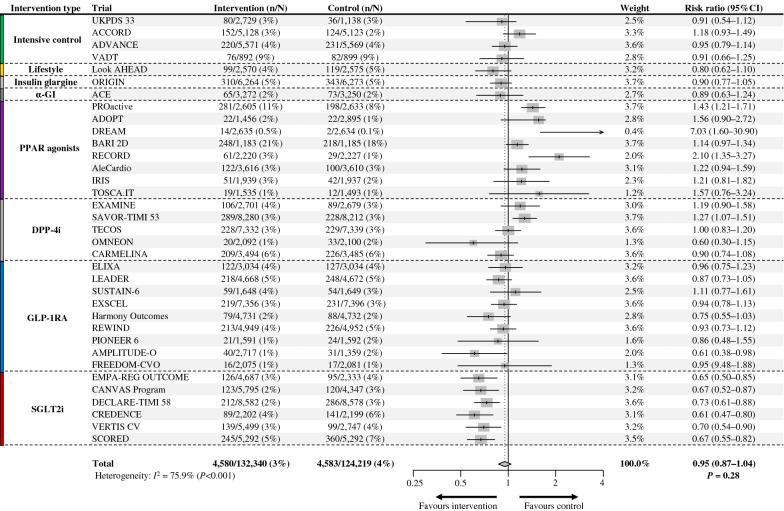
Efficacy of antihyperglycemic drugs on the risk of heart failure (HF). UKPDS 33, ACCORD, ADVANCE, VADT: trials comparing an intensive glycemic control strategy with standard care; Look AHEAD: a trial comparing intensive lifestyle intervention for weight loss with standard care; ORIGIN: a trial comparing insulin glargine with standard care; ACE: a trial comparing acarbose (α-glucosidase inhibitor [α-GI]) with placebo; PROactive, ADOPT, DREAM, BARI 2D, RECORD, AleCardio, IRIS, TOSCA.IT: trials comparing peroxisome proliferation-activated receptor (PPAR) agonists with placebo or active control drug; EXAMINE, SAVOR-TIMI 53, TECOS, OMNEON, CARMELINA: trials comparing dipeptidyl-peptidase-4 inhibitors (DPP-4i) with placebo; ELIXA, LEADER, SUSTAIN-6, EXSCEL, Harmony Outcomes, REWIND, PIONEER 6, AMPLITUDE-O, FREEDOM-CVO: trials comparing glucagon-like peptide-1 receptor agonists (GLP-1RA) with placebo; EMPAREG-OUTCOME, CANVAS-Program, DECLARE-TIMI 58, CREDENCE, VERTIS CV, SCORED: trials comparing sodium-glucose cotransporter-2 inhibitors (SGLT2i) with placebo

Overall, 9163 patients (3.6%) experienced HF outcomes during the follow-up period. In the pooled analysis of 35 trials, antihyperglycemic therapies conferred no overall significant effect on HF risk with high heterogeneity across studies (RR: 0.95; 95% CI 0.87–1.04; *P* = 0.28; *I*^*2*^ = 75.9%) (Fig. 4). In a subgroup analysis based on the type of intervention, GLP-1RA (RR: 0.90; 95% CI 0.83–0.98; *P* = 0.019; *I*^*2*^ = 0.0%) and SGLT2i (RR: 0.68; 95% CI 0.62–0.75; *P* < 0.001; *I*^*2*^ = 0.0%) significantly reduced HF risk with a greater reduction of risk with SGLT2i. PPAR agonists significantly increased HF risk by 38% (RR: 1.38; 95% CI 1.19–1.60; *P* < 0.001; *I*^*2*^ = 53.0%). The others showed neutral effects on HF risk (Additional file 1: Fig. S5). Notably, SGLT2i lowered HF risk more than any other type of intervention, as indicated by the non-overlapping CI.

**Fig. 5:**
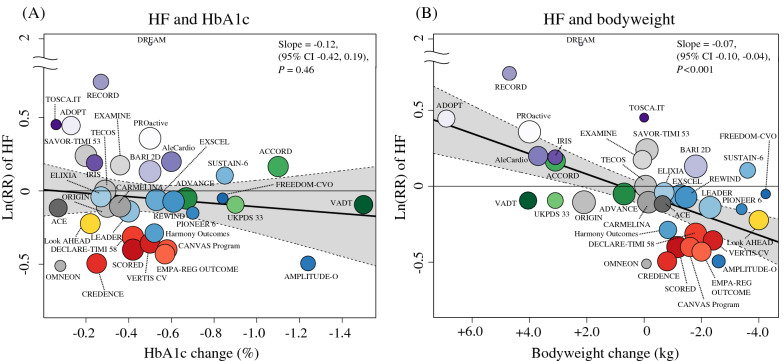
Association between heart failure (HF) risk and **A** HbA1c reduction or **B** bodyweight change. The thicker line shows meta-regression with 95% CI as shading. The circle size of each trial inversely reflects the study weight. *HbA1c* glycated hemoglobin, *CI* confidence interval, *Ln(RR)* estimated log risk ratio

In meta-regression analyses, the Ln(RR) of HF was not significantly associated with HbA1c reduction (slope: − 0.12; 95% CI − 0.42 to 0.19; *P* = 0.46) (Fig. 5A), in contrast to the results from the meta-regression analysis of MACE outcomes. Instead, bodyweight reduction was significantly associated with the Ln(RR) of HF (slope: − 0.07; 95% CI − 0.10 to − 0.04; *P* < 0.001) (Fig. 5B). Accordingly, every 1 kg greater reduction in bodyweight was associated with a 7% (95% CI: 4–10) relative RR reduction of HF. However, significant residual heterogeneity (*P* < 0.001) was observed despite the variance explained (52%) was comparable with the variance explained by HbA1c for MACE (52%). Notably, all trials involving SGLT2i did not align well with the regression slope.

We assessed the consistency of the relationship between bodyweight loss and the risk of HF through subgroup and sensitivity meta-regression analyses. In the stratified meta-regression by type of intervention, trials involving intensive glycemic control strategies, PPAR agonists, DPP-4i, and GLP-1RA showed a trend towards reducing the Ln(RR) of HF in association with a decrease in bodyweight; however, the association between bodyweight reduction and HF risk was not statistically significant for all intervention types (Additional file 1: Table S5). In the stratified meta-regression based on the baseline prevalence of ASCVD (≥ 70% vs. < 70%), bodyweight reduction was significantly associated with a decrease in the Ln(RR) of HF for both subgroups, in line with the overall results (Additional file 1: Fig. S6). In the sensitivity meta-regression analysis with adjustment for multiple confounders (trial-level age, sex, and person-years), bodyweight reduction was significantly associated with the risk reduction of HF consistent with the results of the main univariate analysis (slope: − 0.07; 95% CI − 0.10 to − 0.03; *P* < 0.001; variance explained: 48%).

## Discussion

The current meta-analysis with meta-regression enrolled 256,524 patients with type 2 diabetes or prediabetes from 35 large-scale cardiovascular outcome trials and explored the associations between glycemic improvement or bodyweight change and MACE and HF risks. To our knowledge, this is the largest meta-analysis and meta-regression analysis of randomized controlled trials of antihyperglycemic therapies reporting MACE and HF outcomes. With respect to MACE outcomes, antihyperglycemic therapies significantly reduced MACE risk in the random-effects model meta-analysis, and the HbA1c reduction, not bodyweight reduction, was significantly correlated with a decline in the Ln(RR) of MACE in the univariate and multivariate meta-regression analyses; every 1% additional reduction in HbA1c was associated with a 14% relative reduction in MACE risk. The subgroup analysis further demonstrated that the relationship between HbA1c reduction and MACE risk reduction was more pronounced in patients with advanced atherosclerosis. These findings indicated potential effect modifications of MACE outcomes through glycemic control and reiterated the utility of HbA1c reduction as a marker of MACE risk reduction.

Regarding HF, antihyperglycemic therapies demonstrated a trend towards reducing HF risk compared with controls, with a RR of 0.95 (95% CI 0.87–1.04) in the meta-analysis, whereas the results showed high heterogeneity across trials. Contrary to MACE, bodyweight change, not HbA1c reduction, was associated with the Ln(RR) of HF in the univariate and multivariate meta-regression analyses. However, residual heterogeneity was high, and all trials involving SGLT2i did not align well with the regression slope; these findings indicated that while bodyweight reduction can partially contribute to HF risk reduction, other factors still influence HF risk modulation. In particular, the data on SGLT2i suggest a greater influence of residual contributors beyond the reduction in bodyweight and HbA1c on HF risk amelioration.

### Glycemic control, bodyweight reduction, and major adverse cardiovascular events

In our study, antihyperglycemic therapies reduced MACE risk by 9%, which is almost identical to the MACE risk reduction reported by the previous meta-analyses of cardiovascular outcome trials with intensive glycemic control [54] and those with newer antihyperglycemic medications conducted after the establishment of the FDA guidelines in 2008 [5]. The significant association between HbA1c decline and attenuation of the MACE risk indicates that blood glucose lowering would proportionally decrease the risk of MACE, in agreement with the observations of the post hoc studies of the LEADER and REWIND trials, which suggested that HbA1c was a major and significant mediator of the cardiovascular benefits [55, 56]. Moreover, the findings in those mediation analyses that bodyweight was not a significant mediator of cardiovascular benefits for GLP-1RA are consistent with the non-significant association between bodyweight change and MACE risk in our meta-regression analysis [54, 55]. Additionally, most of the cardiovascular benefits associated with SGLT2i were presumed to be attributed to HbA1c reduction in three large cardiovascular outcome trials (EMPA-REG OUTCOME, CANVAS Program, and DECLARE-TIMI 58 trials) [57]. The previous meta-analysis results also corroborate the significant association between glycemic improvement and reduced risk of MACE [4–7]. Our finding supports the hypothesis that HbA1c reduction can be a useful clinical marker of MACE risk reduction across a wide range of antihyperglycemic therapies. Although high hypoglycemia risk associated with antihyperglycemic therapies may dilute the cardiovascular benefit conferred by glycemic reduction [58], blood glucose lowering remains a crucial aspect of cardiovascular risk management and is likely to contribute significantly to reducing MACE risk, as supported by a recent causal directed acyclic graphs study [7]. However, we note that the observed HbA1c reduction can be a marker representing multiple factors, including pleiotropic effects, rather than a single marker of improvement in glycemic control. Pleiotropic effects may include mitigation of endothelial dysfunction and oxidative stress, as observed with GLP-1RA and SGLT2i administration [59, 60]. Further studies are required to disentangle the contribution of glycemic and non-glycemic effects.

### Glycemic control, bodyweight reduction, and heart failure

Regarding HF, antihyperglycemic therapies numerically but not significantly reduced HF risk by 5%, and both primary analysis and subgroup analysis showed high heterogeneity across studies. Contrary to MACE risk, HbA1c reduction was not associated with HF risk. Subgroup analyses show differing effects on HF based on the type of intervention, with SGLT2i and GLP-1RA reducing risk and PPAR agonists increasing it. This suggests glycemic control may not be critical for short-term (< 10 years) prevention or treatment of HF in dysglycemia.

In agreement with our meta-regression analysis results, the previous meta-regression analysis results showed that bodyweight reduction was significantly associated with a reduced HF risk [3]. This is theoretically reasonable because the two drugs that reduced HF risk (SGLT2i and GLP-1RA) decrease bodyweight through specific mechanisms [61], and PPAR agonists, which increased HF risk, increase bodyweight via fluid retention [62]. Given the favorable hemodynamic effects, such as ameliorated high blood pressure and fluid congestion, associated with bodyweight loss [63], the significant association we discovered between bodyweight loss and decreased risk of HF is biologically plausible. However, high residual heterogeneity (*P* < 0.001) and disproportionate reduction of HF risk by SGLT2i (Fig. 5B) suggest the involvement of other important factors in reducing HF risk. Considering that multiple post hoc analyses of the trials involving SGLT2i revealed that changes in markers of volume status and hemoconcentration (e.g., hematocrit), but not in bodyweight, are the most important mediators of cardiovascular death and HF, the clinical markers of the plasma volume status might be the more reliable markers of HF benefits [64–67]. This hypothesis is supported by an observational study reporting that lower hematocrit levels are associated with an increased risk of hospitalization for patients with HF [68].

### Strengths and weaknesses

The main strength of our study is the inclusion of the largest number of cardiovascular outcome trials investigating various antihyperglycemic therapies conducted both before and after the establishment of the FDA guidelines in 2008, thereby allowing the most comprehensive evaluation of the contribution of blood glucose and bodyweight control to MACE and HF risks. Our study has important clinical implications–we highlight the utility of HbA1c and bodyweight changes as useful surrogates for cardiovascular and HF benefits, respectively, and also show class-specific benefits of SGLT2i beyond HbA1c or bodyweight reduction.

However, this study has several limitations. First, we did not use individual participant data, thus precluding our ability to adjust for some potential confounders. A meta-analysis performed with individual participant data could illustrate the independent effect of potential mediators of HF risk reduction, such as plasma volume, vascular resistance, and ketone bodies [59, 60, 64, 69]. Second, we did not evaluate the relationship of MACE or HF with conventional cardiovascular risks such as hypertension [70] and dyslipidemia [71] due to the limited availability of these data. Third, the included trials varied in their design, population, controls, and definitions of MACE and HF outcomes (Table 1, Additional file 1: Table S1); therefore, the pooled effects have to be interpreted with caution. However, the inclusion of a wider variety of trials, many of which represent the basis for the international clinical practice guidelines, allowed for more robust insights into the relationship between HbA1c or bodyweight change and cardiovascular outcomes. Fourth, it is essential to exercise caution in interpreting the results of the stratified meta-regression by type of intervention (Additional file 1: Tables S4 and S5), as a limited number of trials in each subgroup analysis increases the risk of overfitting and magnifies the variability of individual trial results, including any random error.

## Conclusions

The updated meta-analysis and meta-regression analysis of 35 cardiovascular outcome trials show that glycemic control conferred by a wide range of antihyperglycemic drugs decreases MACE risk in an HbA1c-dependent manner, and the degree of HbA1c reduction is a useful surrogate of cardiovascular benefits. Contrary to MACE risk reduction, HF risk modulation was not associated with HbA1c reduction but was associated with bodyweight reduction. However, high residual heterogeneity suggests the contributions of other factors. Importantly, SGLT2 inhibitors reduced the risk of HF more than could be explained by HbA1c or bodyweight reduction, highlighting the drug class-specific benefits for HF.

## Supplementary Information

**Additional file 1: Table S1.** Definition of heart failure and major adverse cardiovascular events of the included trials. **Table S2.** Excluded trials through detailed full-text assessment. **Table S3.** Risk of bias of the included trials. **Table S4.** Univariate meta-regression analyses of HbA1c reduction and the estimated log risk ratio of major adverse cardiovascular events based on intervention type. **Table S5.** Univariate meta-regression analyses of bodyweight change and the estimated log risk ratio of heart failure based on intervention type. **Figure S1.** Derivation of formula to obtain the relative risk ratio reduction of outcomes with meta-regression results. **Figure S2.** Funnel plots for assessing publication bias of major adverse cardiovascular events (MACE) and heart failure (HF) outcomes. **Figure S3.** Efficacy of antihyperglycemic therapies on the risk of major adverse cardiovascular events (MACE) in each subgroup. **Figure S4.** Association between the risk of major adverse cardiovascular events (MACE) and HbA1c reduction stratified by the baseline prevalence of ASCVD. **Figure S5.** Efficacy of antihyperglycemic therpies on the risk of heart failure (HF) in each subgroup. **Figure S6.** Association between heart failure (HF) risk and bodyweight change stratified by the baseline prevalence of ASCVD.

## Data Availability

All data were extracted from publicly available sources and are included in this published article and its Additional file.

## Abbreviations

ASCVD: Atherosclerotic cardiovascular disease
CI: Confidence interval
CVOTs: Cardiovascular outcome trials
DPP-4i: Dipeptidyl-peptidase-4 inhibitors
Ln(RR): Estimated log risk ratio
FDA: Food and Drug Administration
GLP-1RA: Glucagon-like receptor agonists
HbA1c: Glycated hemoglobin
HF: Heart failure
MACE: Major adverse cardiovascular events
MI: Myocardial infarction
PPAR: Peroxisome proliferation-activated receptor
PRISMA: Preferred Reporting Items for Systematic Reviews and Meta-analysis
RR: risk ratio
SGLT2i: Sodium-glucose cotransporter-2 inhibitors
UKPDS: United Kingdom Prospective Diabetes Study
