# Correction: Efficacy of antihyperglycemic therapies on cardiovascular and heart failure outcomes: an updated meta-analysis and meta-regression analysis of 35 randomized cardiovascular outcome trials

**DOI:** 10.1186/s12933-023-01825-4

**Published:** 2023-04-24

**Authors:** Masashi Hasebe, Satoshi Yoshiji, Yamato Keidai, Hiroto Minamino, Takaaki Murakami, Daisuke Tanaka, Yoshihito Fujita, Norio Harada, Akihiro Hamasaki, Nobuya Inagaki

**Affiliations:** 1 Department of Diabetes and Endocrinology, Medical Research Institute KITANO HOSPITAL, PIIF Tazuke-Kofukai, Osaka, Japan; 2 Department of Diabetes, Endocrinology and Nutrition, Kyoto University Graduate School of Medicine, 54 Kawahara‑cho, Shogoin, Sakyo‑ku, Kyoto, 606‑8507 Japan; 3 Department of Human Genetics, McGill University, Montreal, QC Canada; 4 Kyoto‑McGill International Collaborative Program in Genomic Medicine, Graduate School of Medicine, Kyoto University, Kyoto, Japan

**Correction: Cardiovascular Diabetology (2023) 22:62** 10.1186/s12933-023-01773-z

Following the publication of the original article [1], the authors identified errors in Fig. 2 introduced during the final production phase. Specifically, the sample size numbers in the rows for 'Look AHEAD,' 'ADOPT,' 'BARI2D,' 'RECORD,' 'TOSCA.IT,' and 'Total' were garbled. The results were not affected. The corrected Fig. 2 has been provided with this correction.

**Fig. 2:**
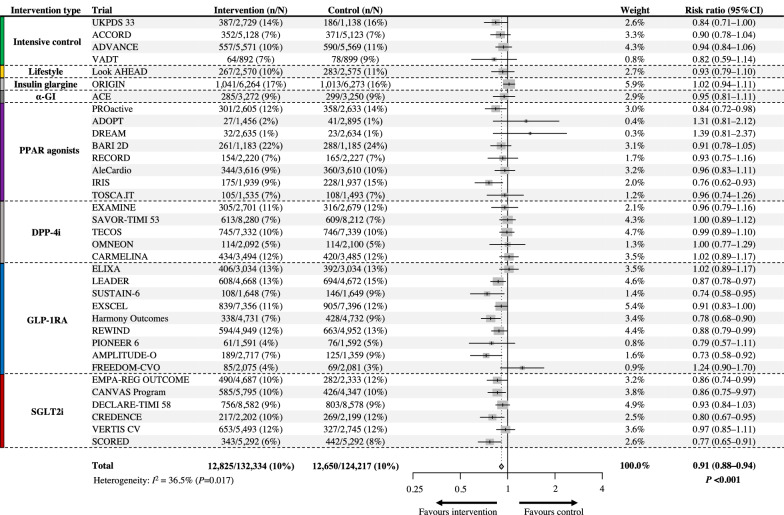
Efficacy of antihyperglycemic drugs on the risk of major adverse cardiovascular events (MACE). UKPDS 33, ACCORD, ADVANCE, VADT: trials comparing an intensive glycemic control strategy with standard care; Look AHEAD: a trial comparing intensive lifestyle intervention for weight loss with standard care; ORIGIN: a trial comparing insulin glargine with standard care; ACE: a trial comparing acarbose (α-glucosidase inhibitor [α-GI]) with placebo; PROactive, ADOPT, DREAM, BARI 2D, RECORD, AleCardio, IRIS, TOSCA.IT: trials comparing peroxisome proliferation-activated receptor (PPAR) agonists with placebo or active control drug; EXAMINE, SAVOR-TIMI 53, TECOS, OMNEON, CARMELINA: trials comparing dipeptidyl-peptidase-4 inhibitors (DPP-4i) with placebo; ELIXA, LEADER, SUSTAIN-6, EXSCEL, Harmony Outcomes, REWIND, PIONEER 6, AMPLITUDE-O, FREEDOM-CVO: trials comparing glucagon-like peptide-1 receptor agonists (GLP-1RA) with placebo; EMPAREG-OUTCOME, CANVAS-Program, DECLARE-TIMI 58, CREDENCE, VERTIS CV, SCORED: trials comparing sodium-glucose cotransporter-2 inhibitors (SGLT2i) with placebo

The original article has been updated.
