# Supplementary material for: Efficacy of antihyperglycemic therapies on cardiovascular and heart failure outcomes: an updated meta-analysis and meta-regression analysis of 35 randomized cardiovascular outcome trials
Source: Cardiovasc Diabetol. 2023 Mar 19;22:62. doi: 10.1186/s12933-023-01773-z (PMC10024854; doi:10.1186/s12933-023-01773-z)
Supplement: Supplementary file 1 — Additional file 1: Table S1. Definition of heart failure and major adverse cardiovascular events of the included trials. Table S2. Excluded trials through detailed full-text assessment. Table S3. Risk of bias of the included trials. Table S4. Univariate meta-regression analyses of HbA1c reduction and the estimated log risk ratio of major adverse cardiovascular events based on intervention type. Table S5. Univariate meta-regression analyses of bodyweight change and the estimated log risk ratio of heart failure based on intervention type. Figure S1. Derivation of formula to obtain the relative risk ratio reduction of outcomes with meta-regression results. Figure S2. Funnel plots for assessing publication bias of major adverse cardiovascular events (MACE) and heart failure (HF) outcomes. Figure S3. Efficacy of antihyperglycemic therapies on the risk of major adverse cardiovascular events (MACE) in each subgroup. Figure S4. Association between the risk of major adverse cardiovascular events (MACE) and HbA1c reduction stratified by the baseline prevalence of ASCVD. Figure S5. Efficacy of antihyperglycemic therpies on the risk of heart failure (HF) in each subgroup. Figure S6. Association between heart failure (HF) risk and bodyweight change stratified by the baseline prevalence of ASCVD. [file 12933_2023_1773_MOESM1_ESM.pdf]

Supplemental Materials

Efficacy of antihyperglycemic therapies on cardiovascular and heart failure outcomes: An updated meta-analysis and meta-regression analysis of 35 randomized cardiovascular outcome trials

Masashi Hasebe, M.D., Satoshi Yoshiji, M.D.,Yamato Keidai, M.D., Hiroto Minamino, M.D., Takaaki Murakami, M.D., Ph.D., Daisuke Tanaka, M.D., Ph.D., Yoshihito Fujita, M.D., Ph.D., Norio Harada, M.D., Ph.D., Akihiro Hamasaki, M.D., Ph.D., Nobuya Inagaki, M.D., Ph.D.

Table S1. Definition of heart failure and major adverse cardiovascular events of the included trials
Table S2. Excluded trials through detailed full-text assessment
Table S3. Risk of bias of the included trials
Table S4. Univariate meta-regression analyses of HbA1c reduction and the estimated log risk ratio of major adverse cardiovascular events based on intervention type
Table S5. Univariate meta-regression analyses of bodyweight change and the estimated log risk ratio of heart failure based on intervention type

Figure S1. Derivation of formula to obtain the relative risk ratio reduction of outcomes with meta-regression results
Figure S2. Funnel plots for assessing publication bias of major adverse cardiovascular events (MACE) and heart failure (HF) outcomes
Figure S3. Efficacy of antihyperglycemic therapies on the risk of major adverse cardiovascular events (MACE) in each subgroup
Figure S4. Association between the risk of major adverse cardiovascular events (MACE) and HbA1c reduction stratified by the baseline prevalence of ASCVD
Figure S5. Efficacy of antihyperglycemic therpies on the risk of heart failure (HF) in each subgroup
Figure S6. Association between heart failure (HF) risk and bodyweight change stratified by the baseline prevalence of ASCVD

Table S1. Definition of heart failure and major adverse cardiovascular events of the included trials

Trial	Year	HF definition	MACE definition
UKPDS 33	1998	HF (not associated with MI), by clinical symptoms confirmed by Kerley B lines, rales, raised jugular venous pressure, or third heart sound	Fatal and non-fatal MI
PROactive	2005	HF not requiring or requiring hospitalization or prolonged a hospitalization stay, was fatal or life threatening, or resulted in persistent significant disability or incapacity	All-cause death, non-fatal MI (excluding silent), or non-fatal stroke
ADOPT	2006	Investigator-reported congestive HF	Fatal and non-fatal MI
DREAM	2006	Acute treatment with at least two of the following criteria: typical signs and symptoms, typical radiological evidence, use of diuretics, vasodilators, or inotropes	CV death, non-fatal MI, or non-fatal stroke
ACCORD	2008	Fatal or non-fatal congestive HF	CV death, non-fatal MI, or non-fatal stroke
ADVANCE	2008	Death due to heart failure, hospitalization for heart failure, or worsening New York Heart Association class	CV death, non-fatal MI, or non-fatal stroke
BARI 2D	2009	The manifestation of one or more symptoms, including dyspnea upon exertion, bilateral pedal edema, fatigue, orthopnea, and paroxysmal nocturnal dyspnea	All-cause death, non-fatal MI, or non-faral stroke
RECORD	2009	HF resulting in death or non-fatal HF requiring hospitalization	CV death, non-fatal MI, or non-fatal stroke
VADT	2009	New or worsening congestive HF	CV death, non-fatal MI, or non-fatal stroke
ORIGIN	2012	HF requiring hospitalization	CV death, non-fatal MI, or non-fatal stroke
EXAMINE	2013	HF requiring hospitalization	CV death, non-fatal MI, or non-fatal stroke
Look AHEAD	2013	Congestive HF requiring hospitalization	CV death, non-fatal MI, or non-fatal stroke
SAVOR-TIMI 53	2013	HF requiring hospitalization	CV death, non-fatal MI, or non-fatal ischemic stroke
AleCardio	2014	HF requiring hospitalization	CV death, non-fatal MI, or non-fatal stroke
ELIXA	2015	HF requiring hospitalization	CV death, non-fatal MI, non-fatal stroke, or hospitalization for unstable angina
EMPA-REG OUTCOME	2015	HF requiring hospitalization	CV death, non-fatal MI (excluding silent) , or non-fatal stroke
TECOS	2015	HF requiring hospitalization	CV death, non-fatal MI, or non-fatal stroke
IRIS	2016	HF requiring hospitalization or resulting in death	Fatal and non-fatal MI or stroke
LEADER	2016	HF requiring hospitalization	CV death, non-fatal MI (including silent) , or non-fatal stroke
SUSTAIN-6	2016	HF requiring hospitalization	CV death, non-fatal MI, or non-fatal stroke
OMNEON	2017	HF requiring hospitalization	CV death, non-fatal MI, or non-fatal stroke
CANVAS Program	2017	HF requiring hospitalization	CV death, non-fatal MI, or non-fatal stroke
EXSCEL	2017	HF requiring hospitalization	CV death, non-fatal MI, or non-fatal stroke
ACE	2017	HF requiring hospitalization	CV death, non-fatal MI, or non-fatal stroke
TOSCA.IT	2017	HF requiring hospitalization and other outpatient escalation in care	All-cause death, non-fatal MI (including silent), non-fatal stroke, or urgent coronary revascularization
Harmony Outcomes	2018	HF requiring hospitalization	CV death, non-fatal MI, or non-fatal stroke
DECLARE-TIMI 58	2019	HF requiring hospitalization	CV death, non-fatal MI, or non-fatal ischemic stroke
CARMELINA	2019	HF requiring hospitalization	CV death, non-fatal MI, or non-fatal stroke
CREDENCE	2019	HF requiring hospitalization	CV death, non-fatal MI, or non-fatal stroke
REWIND	2019	HF requiring hospitalization or urgent visit	CV death (including death from unknown causes), non-fatal MI, or non-fatal stroke
PIONEER 6	2019	HF requiring hospitalization	CV death, non-fatal MI, or non-fatal stroke
VERTIS CV	2020	HF requiring hospitalization	CV death, non-fatal MI, or non-fatal stroke
SCORED	2021	HF requiring hospitalization or urgent visit	CV death, non-fatal MI, or non-fatal stroke
AMPLITUDE-O	2021	HF requiring hospitalization	CV death (including death from undetermmined causes), non-fatal MI, or non-fatal stroke
FREEDOM-CVO	2022	HF requiring hospitalization	CV death, non-fatal MI, or non-fatal stroke

Abbreviations: HF, heart failure; MACE, major adverse cardiovascular events; MI, myocardial infarction; CV death, cardiovascular death.

Table S2. Excluded trials through detailed full-text assessment

Trial	Year	Excluded reason	Reference
Davey Smith G, et al	2005	enrolled patients without dysglycemia and not reporting an outcome of interest	Davey Smith G, Bracha Y, Svendsen KH, et al. Incidence of type 2 diabetes in the randomized multiple risk factor intervention trial. *Ann Intern Med*. 2005;142(5):313–322.
Japan Diabetes Complications Study	2010	not reporting an outcome of interest	Sone H, Tanaka S, Iimuro S, et al. Long-term lifestyle intervention lowers the incidence of stroke in Japanese patients with type 2 diabetes: a nationwide multicentre randomised controlled trial (the Japan Diabetes Complications Study). *Diabetologia*. 2010;53(3):419–428.
CAROLINA	2019	HbA1c difference between trial arms ≤0.01%	Rosenstock J, Kahn SE, Johansen OE, ey al. Effect of linagliptin vs glimepiride on major adverse cardiovascular outcomes in patients with type 2 diabetes: The CAROLINA randomized clinical trial. *JAMA*. 2019;322(12):1155–1166.
ANDREW	2020	non-randomized controlled trial without reporting an outcome of interest	Bossi AC, De Mori V, Scaranna C, et al. ANDREW: A multicenter, prospective, observational study in patients with type 2 diabetes on persistent treatment with dulaglutide. *Diabetes Ther*. 2020;11(11):2677–2690.
SOLOIST-WHF	2021	ended early due to loss of funding from the sponsor with duration of <1 year	Bhatt DL, Szarek M, Steg PG, et al. Sotagliflozin in patients with diabetes and recent worsening heart failure. *N Engl J Med*. 2021;384(2):117–128.
Tuttolomondo A, et al.	2021	enrolled less than 1,000 patients (*n* = 124)	Tuttolomondo A, Cirrincione A, Casuccio A, et al. Efficacy of dulaglutide on vascular health indexes in subjects with type 2 diabetes: a randomized trial. *Cardiovasc Diabetol*. 2021;20(1):1.
SURPASS-4	2021	not reporting HbA1c difference between an intervention (pooled tirzepatide groups) and a control (insulin glargine)	Del Prato S, Kahn SE, Pavo I, et al. Tirzepatide versus insulin glargine in type 2 diabetes and increased cardiovascular risk (SURPASS-4): a randomised, open-label, parallel-group, multicentre, phase 3 trial. *Lancet*. 2021;398(10313):1811–1824.
ACADEMIC	2022	short duration of <1 year	Gao B, Gao W, Wan H, et al. Efficacy and safety of alogliptin versus acarbose in Chinese type 2 diabetes patients with high cardiovascular risk or coronary heart disease treated with aspirin and inadequately controlled with metformin monotherapy or drug-naive: A multicentre, randomized, open-label, prospective study (ACADEMIC). *Diabetes Obes Metab*. 2022 :24(6):991–999.
GRADE	2022	not reporting an outcome of interest	Nathan DM, Lachin JM, Balasubramanyam A, et al. Glycemia reduction in type 2 diabetes - glycemic outcomes. *N Engl J Med*. 2022;387(12):1063–1074.

Table S3. Risk of bias of the included trials

Trial	Year	Randomization process	Deviations from intended interventions	Missing outcome data	Measurement of the outcome	Selection of the reported result	Overall bias
UKPDS 33	1998	Low risk	High risk	Low risk	Low risk	Low risk	Low risk
PROactive	2005	Low risk	Low risk	Low risk	Low risk	Low risk	Low risk
ADOPT	2006	Low risk	Low risk	Low risk	Low risk	Low risk	Low risk
DREAM	2006	Low risk	Low risk	Low risk	Low risk	Low risk	Low risk
ACCORD	2008	Low risk	High risk	Low risk	Low risk	Low risk	Low risk
ADVANCE	2008	Low risk	High risk	Low risk	Low risk	Low risk	Low risk
BARI 2D	2009	Low risk	High risk	Low risk	Low risk	Low risk	Low risk
RECORD	2009	Low risk	High risk	Low risk	Low risk	Low risk	Low risk
VADT	2009	Low risk	High risk	Low risk	Low risk	Low risk	Low risk
ORIGIN	2012	Low risk	High risk	Low risk	Some concerns	Low risk	Low risk
EXAMINE	2013	Low risk	Low risk	Low risk	Low risk	Low risk	Low risk
Look AHEAD	2013	Low risk	High risk	Low risk	Low risk	Low risk	Low risk
SAVOR-TIMI 53	2013	Low risk	Low risk	Low risk	Low risk	Low risk	Low risk
AleCardio	2014	Low risk	Low risk	Low risk	Low risk	Low risk	Low risk
ELIXA	2015	Low risk	Low risk	Low risk	Low risk	Low risk	Low risk
EMPA-REG OUTCOME	2015	Low risk	Low risk	Low risk	Low risk	Low risk	Low risk
TECOS	2015	Low risk	Low risk	Low risk	Low risk	Low risk	Low risk
IRIS	2016	Low risk	Low risk	Low risk	Low risk	Low risk	Low risk
LEADER	2016	Low risk	Low risk	Low risk	Low risk	Low risk	Low risk
SUSTAIN-6	2016	Low risk	Low risk	Low risk	Low risk	Low risk	Low risk
OMNEON	2017	Low risk	Low risk	Low risk	Low risk	Low risk	Low risk
CANVAS Program	2017	Low risk	Low risk	Low risk	Low risk	Low risk	Low risk
EXSCEL	2017	Low risk	Low risk	Low risk	Low risk	Low risk	Low risk
ACE	2017	Low risk	Low risk	Low risk	Low risk	Low risk	Low risk
TOSCA.IT	2017	Low risk	High risk	Low risk	Low risk	Low risk	Low risk
Harmony Outcomes	2018	Low risk	Low risk	Low risk	Low risk	Low risk	Low risk
DECLARE-TIMI 58	2019	Low risk	Low risk	Low risk	Low risk	Low risk	Low risk
CARMELINA	2019	Low risk	Low risk	Low risk	Low risk	Low risk	Low risk
CREDENCE	2019	Low risk	Low risk	Low risk	Low risk	Low risk	Low risk
REWIND	2019	Low risk	Low risk	Low risk	Low risk	Low risk	Low risk
PIONEER 6	2019	Low risk	Low risk	Low risk	Low risk	Low risk	Low risk
VERTIS CV	2020	Low risk	Low risk	Low risk	Low risk	Low risk	Low risk
SCORED	2021	Low risk	Low risk	Low risk	Low risk	Low risk	Low risk
AMPLITUDE-O	2021	Low risk	Low risk	Low risk	Low risk	Low risk	Low risk
FREEDOM-CVO	2022	Low risk	Low risk	Low risk	Low risk	Low risk	Low risk

Table S4. Univariate meta-regression analyses of HbA1c reduction and the estimated log risk ratio of major adverse cardiovascular events based on intervention type

	Regression coefficient (95% CI)	*P* value
Intensive glycemic control	-0.14 (-0.47 to 0.19)	0.42
PPAR agonists	0.04 (-0.41 to 0.49)	0.85
DPP-4 inhibitors	-0.01 (-1.00 to 0.99)	0.99
GLP-1 receptor agonists	-0.24 (-0.51 to 0.03)	0.076
SGLT-2 inhibitors	0.23 (-0.47 to 0.92)	0.52

Abbreviations: HbA1c, glycated hemoglobin; CI, confidence interval; PPAR agonists, peroxisome proliferation-activated receptor agonists; DPP-4 inhibitors, dipeptidyl-peptidase-4 inhibitors; GLP-1 receptor agonists, glucagon-like peptide-1 receptor agonists; SGLT-2 inhibitors, sodium-glucose cotranspoerter-2 inhibitors.

Table S5. Univariate meta-regression analyses of bodyweight change and the estimated log risk ratio of heart failure based on intervention type

	Regression coefficient (95% CI)	*P* value
Intensive glycemic control	-0.01 (-0.47 to 0.19)	0.81
PPAR agonists	-0.02 (-0.07 to 0.04)	0.56
DPP-4 inhibitors	-0.93 (-3.55 to 1.69)	0.48
GLP-1 receptor agonists	-0.01 (-0.10 to 0.11)	0.90
SGLT-2 inhibitors	0.15 (-0.79 to 1.10)	0.75

Abbreviations: CI, confidence interval; PPAR agonists, peroxisome proliferation-activated receptor agonists; DPP-4 inhibitors, dipeptidyl-peptidase-4 inhibitors; GLP-1 receptor agonists, glucagon-like peptide-1 receptor agonists; SGLT-2 inhibitors, sodium-glucose cotranspoerter-2 inhibitors.

Figure S1. Derivation of formula to obtain the relative risk ratio (RR) reduction of outcomes with meta-regression results

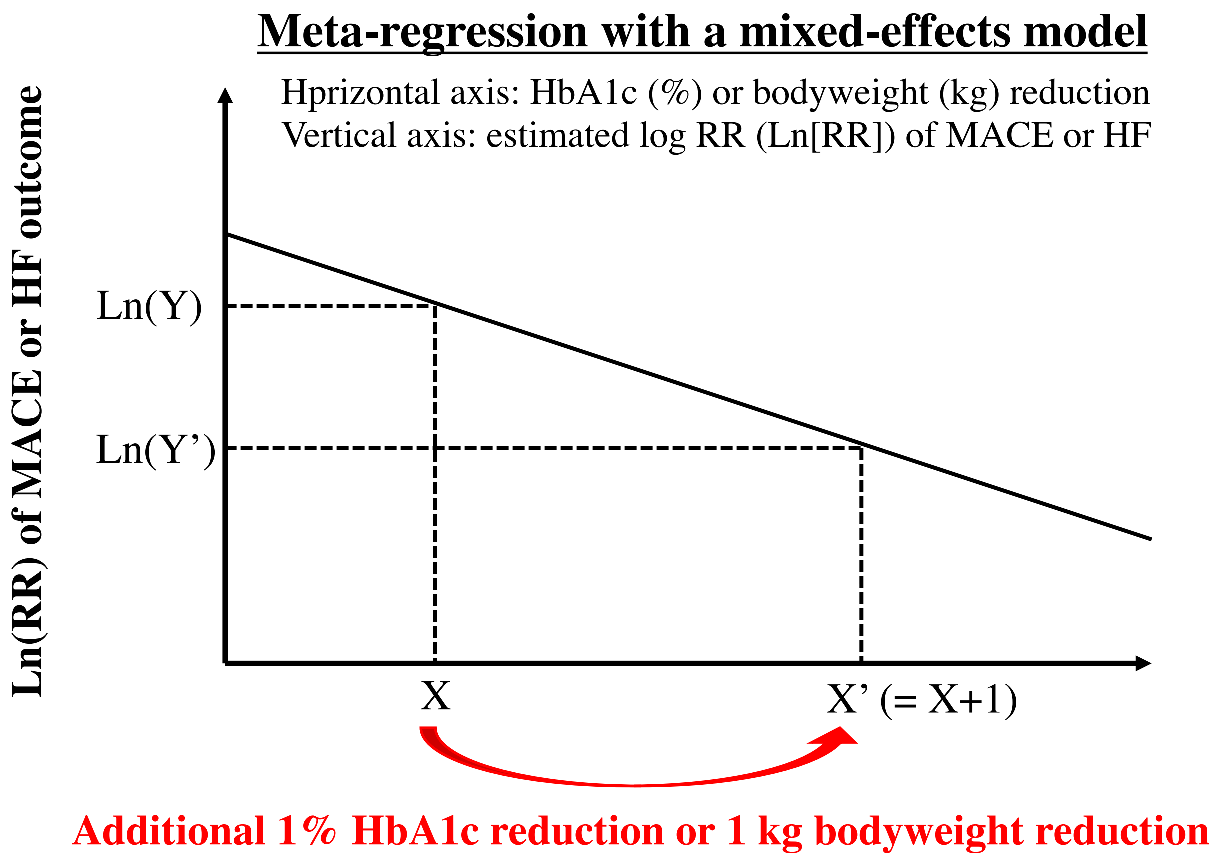

$$\mathrm{Ln}(Y') - \mathrm{Ln}(Y) = \text{regression coefficient (slope)}$$
$$\Leftrightarrow \mathrm{Ln}(Y'/Y) = \text{slope}$$
$$\Leftrightarrow Y'/Y = e^{\text{slope}}$$

∴ Relative RR reduction (%) for every 1% HbA1c reduction or every 1 kg bodyweight reduction

$$= (1 - Y'/Y) \times 100$$
$$= (1 - e^{\text{slope}}) \times 100$$

Y: RR of MACE or HF outcome at a reduction of HbA1c (%) or bodyweight (kg) of X

Y': RR of MACE or HF outcome for a 1% greater HbA1c or a 1 kg greater bodyweight reduction from X (RR at a reduction of HbA1c [%] or bodyweight [kg] of X')

Abbreviations: HbA1c, glycated hemoglobin; MACE, major adverse cardiovascular events; HF, heart failure.

Figure S2. Funnel plots for assessing publication bias of major adverse cardiovascular events (MACE) and heart failure (HF) outcomes

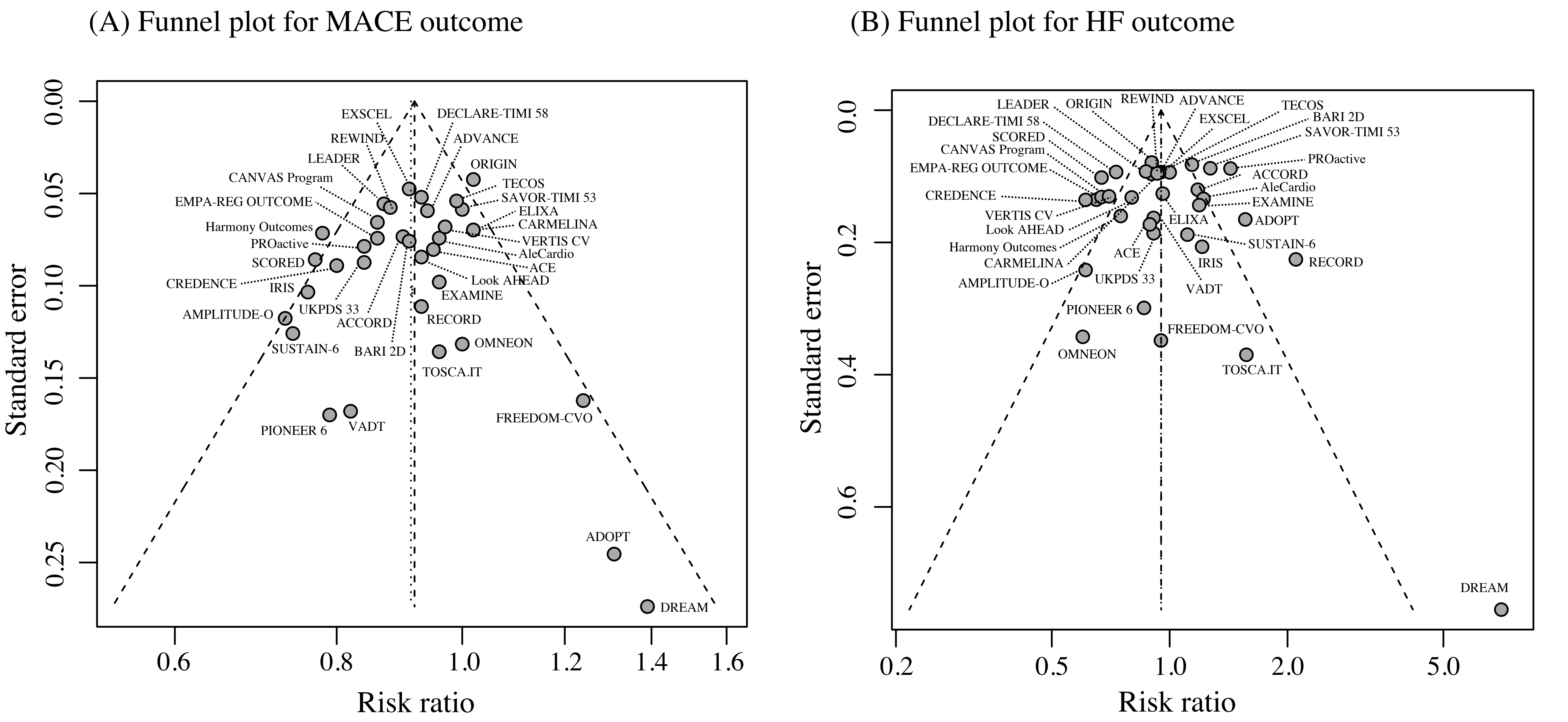

Figure S3. Efficacy of antihyperglycemic therapies on the risk of major adverse cardiovascular events (MACE) in each subgroup

(A) Intensive glycemic control

Trial	Intervention (n/N)	Control (n/N)	Risk ratio (95%CI)
UKPDS 33	387/2,729 (14%)	186/1,138 (16%)	0.84 (0.71–1.00)
ACCORD	352/5,128 (7%)	371/5,123 (7%)	0.90 (0.78–1.04)
ADVANCE	557/5,571 (10%)	590/5,569 (11%)	0.94 (0.84–1.06)
VADT	64/892 (7%)	78/899 (9%)	0.82 (0.59–1.14)
**Total**	**1,360/14,320 (9%)**	**1,225/12,729 (10%)**	**0.90 (0.83–0.97)**
Heterogeneity: $I^2 = 0.0\%$ ($P = 0.69$)			***P* = 0.008**

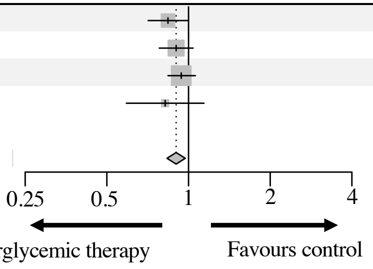

(B) Peroxisome proliferation-activated receptor agonists

Trial	Intervention (n/N)	Control (n/N)	Risk ratio (95%CI)
PROactive	301/2,605 (12%)	358/2,633 (14%)	0.84 (0.72–0.98)
ADOPT	27/1,456 (2%)	41/2,895 (1%)	1.31 (0.81–2.12)
DREAM	32/2,635 (1%)	23/2,634 (1%)	1.39 (0.81–2.37)
BARI 2D	261/1,183 (22%)	288/1,185 (24%)	0.91 (0.78–1.05)
RECORD	154/2,220 (7%)	165/2,227 (7%)	0.93 (0.75–1.16)
AleCardio	344/3,616 (9%)	360/3,610 (10%)	0.96 (0.83–1.11)
IRIS	175/1,939 (9%)	228/1,937 (12%)	0.76 (0.62–0.93)
TOSCA.IT	105/1,535 (7%)	108/1,493 (7%)	0.96 (0.74–1.26)
**Total**	**1,389/17,189 (8%)**	**1,571/18,614 (8%)**	**0.91 (0.84–0.97)**
Heterogeneity: $I^2 = 25.2\%$ ($P = 0.23$)			***P* = 0.006**

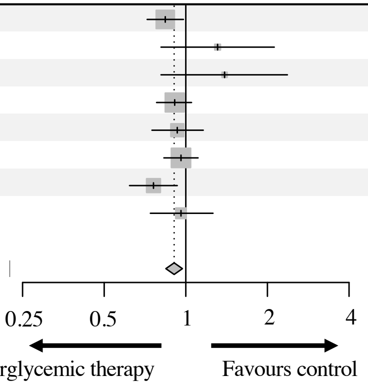

(C) Dipeptidyl-peptidase-4 inhibitors

Trial	Intervention (n/N)	Control (n/N)	Risk ratio (95%CI)
EXAMINE	305/2,701 (11%)	316/2,679 (12%)	0.96 (0.79–1.16)
SAVOR-TIMI 53	613/8,280 (7%)	609/8,212 (7%)	1.00 (0.89–1.12)
TECOS	745/7,332 (10%)	746/7,339 (10%)	0.99 (0.89–1.10)
OMNEON	114/2,092 (5%)	114/2,100 (5%)	1.00 (0.77–1.29)
CARMELINA	434/3,494 (12%)	420/3,485 (12%)	1.02 (0.89–1.17)
**Total**	**2211/23,899 (9%)**	**2205/23,815 (9%)**	**1.00 (0.94–1.06)**
Heterogeneity: $I^2 = 0.0\%$ ($P = 0.99$)			***P* = 0.91**

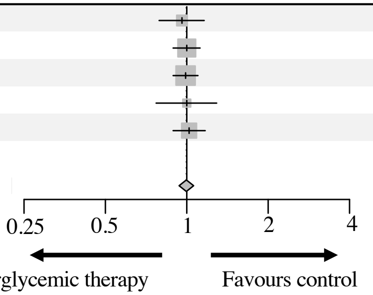

(D) Glucagon-like peptide-1 receptor agonists

Trial	Intervention (n/N)	Control (n/N)	Risk ratio (95%CI)
ELIXA	406/3,034 (13%)	392/3,034 (13%)	1.02 (0.89–1.17)
LEADER	608/4,668 (13%)	694/4,672 (15%)	0.87 (0.78–0.97)
SUSTAIN-6	108/1,648 (7%)	146/1,649 (9%)	0.74 (0.58–0.95)
EXSCEL	839/7,356 (11%)	905/7,396 (12%)	0.91 (0.83–1.00)
Harmony Outcomes	338/4,731 (7%)	428/4,732 (9%)	0.78 (0.68–0.90)
REWIND	594/4,949 (12%)	663/4,952 (13%)	0.88 (0.79–0.99)
PIONEER 6	61/1,591 (4%)	76/1,592 (5%)	0.79 (0.57–1.11)
AMPLITUDE-O	189/2,717 (7%)	125/1,359 (9%)	0.73 (0.58–0.92)
FREEDOM-CVO	85/2,075 (4%)	69/2,081 (3%)	1.24 (0.90–1.70)
**Total**	**3,228/32,769 (10%)**	**3,505/31,467 (11%)**	**0.87 (0.81–0.94)**
Heterogeneity: $I^2 = 53.3\%$ ($P = 0.029$)			***P* = 0.001**

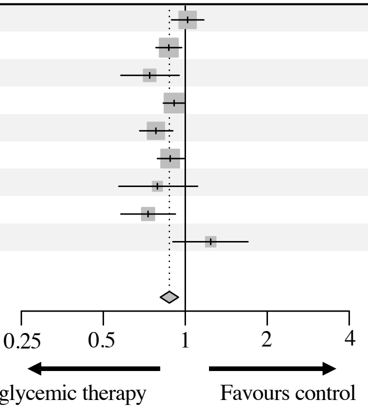

(E) Sodium-glucose cotranspoerter-2 inhibitors

Trial	Intervention (n/N)	Control (n/N)	Risk ratio (95%CI)
EMPA-REG OUTCOME	490/4,687 (10%)	282/2,333 (12%)	0.86 (0.74–0.99)
CANVAS Program	585/5,795 (10%)	426/4,347 (10%)	0.86 (0.75–9.97)
DECLARE-TIMI 58	756/8,582 (9%)	803/8,578 (9%)	0.93 (0.84–1.03)
CREDENCE	217/2,202 (10%)	269/2,199 (12%)	0.80 (0.67–0.95)
VERTIS CV	653/5,493 (12%)	327/2,745 (12%)	0.97 (0.85–1.11)
SCORED	343/5,292 (6%)	442/5,292 (8%)	0.77 (0.65–0.91)
**Total**	**3,044/32,051 (9%)**	**2,549/25,494 (10%)**	**0.88 (0.82–0.94)**
Heterogeneity: $I^2 = 28.1\%$ ($P = 0.22$)			***P* <0.001**

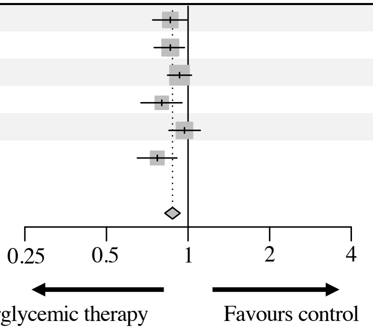

Figure S4. Association between the risk of major adverse cardiovascular events (MACE) and HbA1c reduction stratified by the baseline prevalence of ASCVD

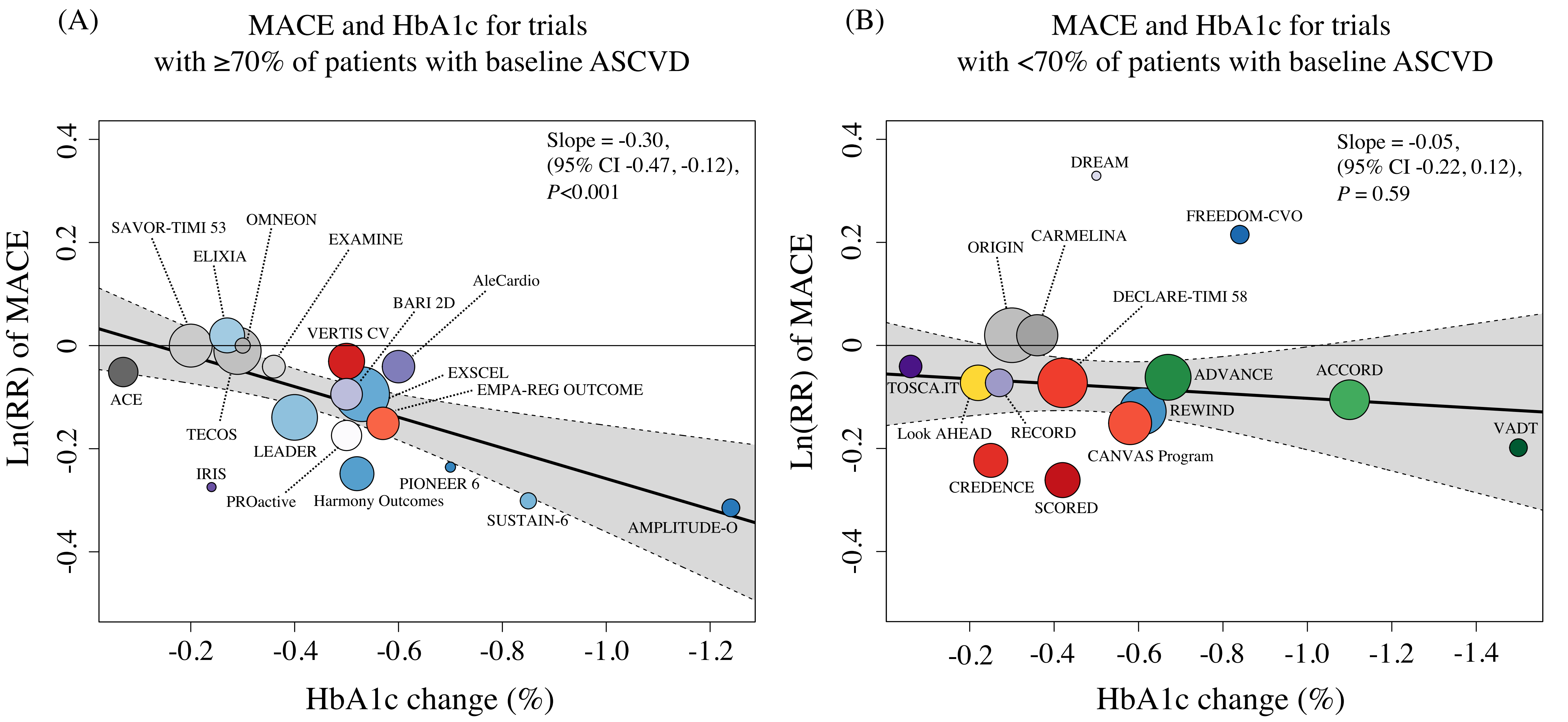

Two trials (UKPDS 33 and ADOPT) without reporting the proportion of patients with baseline ASCVD were excluded from the subgroup analysis.
Abbreviations: ASCVD, atherosclerotic cardiovascular disease; Ln(RR), estimated log risk ratio.

Figure S5. Efficacy of antihyperglycemic therapies on the risk of heart failure (HF) in each subgroup

(A) Intensive glycemic control

Trial	Intervention (n/N)	Control (n/N)	Risk ratio (95%CI)
UKPDS 33	80/2,729 (3%)	36/1,138 (3%)	0.91 (0.54–1.12)
ACCORD	152/5,128 (3%)	124/5,123 (2%)	1.18 (0.93–1.49)
ADVANCE	220/5,571 (4%)	231/5,569 (4%)	0.95 (0.79–1.14)
VADT	76/892 (9%)	82/899 (9%)	0.91 (0.66–1.25)
**Total**	**528/14,320 (4%)**	**473/12,729 (4%)**	**1.00 (0.88–1.14)**
Heterogeneity: $I^2 = 0.0\%$ ($P = 0.43$)			***P* = 0.97**

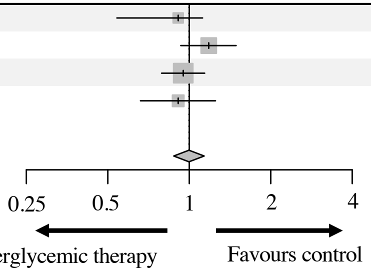

(B) Peroxisome proliferation-activated receptor agonists

Trial	Intervention (n/N)	Control (n/N)	Risk ratio (95%CI)
PROactive	281/2,605 (11%)	198/2,633 (8%)	1.43 (1.21–1.71)
ADOPT	22/1,456 (2%)	22/2,895 (1%)	1.56 (0.90–2.72)
DREAM	14/2,635 (0.5%)	2/2,634 (0.1%)	7.03 (1.60–30.90)
BARI 2D	248/1,183 (21%)	218/1,185 (18%)	1.14 (0.97–1.34)
RECORD	61/2,220 (3%)	29/2,227 (1%)	2.10 (1.35–3.27)
AleCardio	122/3,616 (3%)	100/3,610 (3%)	1.22 (0.94–1.59)
IRIS	51/1,939 (3%)	42/1,937 (2%)	1.21 (0.81–1.82)
TOSCA.IT	19/1,535 (1%)	12/1,493 (1%)	1.57 (0.76–3.24)
**Total**	**818/17189 (5%)**	**623/18614 (3%)**	**1.38 (1.19–1.60)**
Heterogeneity: $I^2 = 53.0\%$ ($P = 0.038$)			***P* <0.001**

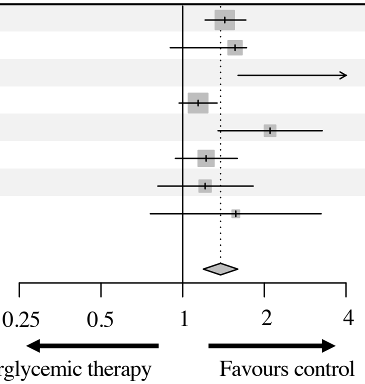

(C) Dipeptidyl-peptidase-4 inhibitors

Trial	Intervention (n/N)	Control (n/N)	Risk ratio (95%CI)
EXAMINE	106/2,701 (4%)	89/2,679 (3%)	1.19 (0.90–1.58)
SAVOR-TIMI 53	289/8,280 (3%)	228/8,212 (3%)	1.27 (1.07–1.51)
TECOS	228/7,332 (3%)	229/7,339 (3%)	1.00 (0.83–1.20)
OMNEON	20/2,092 (1%)	33/2,100 (2%)	0.60 (0.30–1.15)
CARMELINA	209/3,494 (6%)	226/3,485 (6%)	0.90 (0.74–1.08)
**Total**	**852/23,899 (4%)**	**805/23,815 (3%)**	**1.04 (0.88–1.23)**
Heterogeneity: $I^2 = 63.3\%$ ($P = 0.028$)			***P* = 0.63**

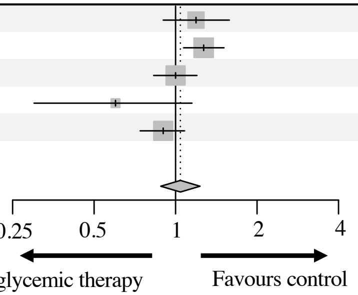

(D) Glucagon-like peptide-1 receptor agonists

Trial	Intervention (n/N)	Control (n/N)	Risk ratio (95%CI)
ELIXA	122/3,034 (4%)	127/3,034 (4%)	0.96 (0.75–1.23)
LEADER	218/4,668 (5%)	248/4,672 (5%)	0.87 (0.73–1.05)
SUSTAIN-6	59/1,648 (4%)	54/1,649 (3%)	1.11 (0.77–1.61)
EXSCEL	219/7,356 (3%)	231/7,396 (3%)	0.94 (0.78–1.13)
Harmony Outcomes	79/4,731 (2%)	88/4,732 (2%)	0.75 (0.55–1.03)
REWIND	213/4,949 (4%)	226/4,952 (5%)	0.93 (0.73–1.12)
PIONEER 6	21/1,591 (1%)	24/1,592 (2%)	0.86 (0.48–1.55)
AMPLITUDE-O	40/2,717 (1%)	31/1,359 (2%)	0.61 (0.38–0.98)
FREEDOM-CVO	16/2,075 (1%)	17/2,081 (1%)	0.95 (0.48–1.88)
**Total**	**974/32,769 (3%)**	**1,046/31,467 (3%)**	**0.90 (0.83–0.98)**
Heterogeneity: $I^2 = 0.0\%$ ($P = 0.66$)			***P* = 0.019**

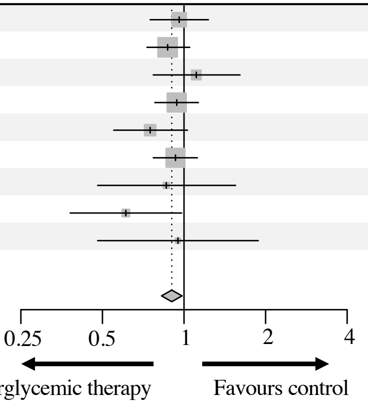

(E) Sodium-glucose cotranspoerter-2 inhibitors

Trial	Intervention (n/N)	Control (n/N)	Risk ratio (95%CI)
EMPA-REG OUTCOME	126/4,687 (3%)	95/2,333 (4%)	0.65 (0.50–0.85)
CANVAS Program	123/57,975 (2%)	120/4,347 (3%)	0.67 (0.52–0.87)
DECLARE-TIMI 58	212/8,582 (2%)	286/8,578 (3%)	0.73 (0.61–0.88)
CREDENCE	89/2,202 (4%)	141/2,199 (6%)	0.61 (0.47–0.80)
VERTIS CV	139/5,499 (3%)	99/2,747 (4%)	0.70 (0.54–0.90)
SCORED	245/5,292 (5%)	360/5,292 (7%)	0.67 (0.55–0.82)
**Total**	**934/32,057 (3%)**	**1101/25,496 (4%)**	**0.68 (0.62–0.75)**
Heterogeneity: $I^2 = 0.0\%$ ($P = 0.92$)			***P* <0.001**

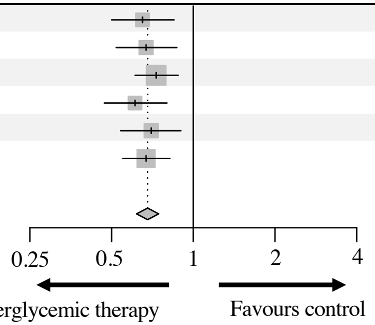

Figure S6. Association between heart failure (HF) risk and bodyweight change stratified by the baseline prevalence of ASCVD

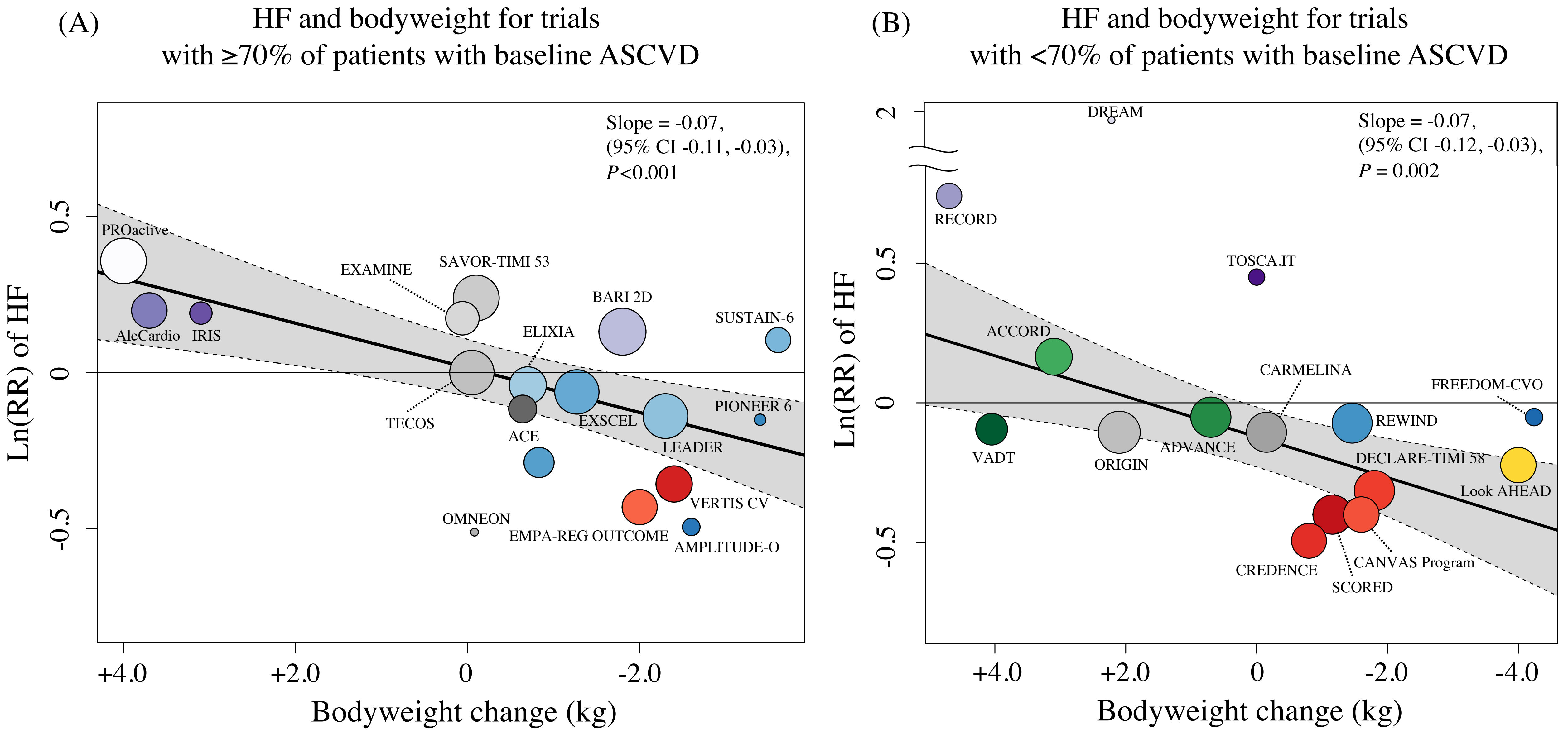

Two trials (UKPDS 33 and ADOPT) without reporting the proportion of patients with baseline ASCVD were excluded from the subgroup analysis.
Abbreviations: ASCVD, atherosclerotic cardiovascular disease; Ln(RR), estimated log risk ratio.
